# Mr. Colman, on an Uncommon Extra-Uterine Fœtus

**Published:** 1799-10

**Authors:** E. Colman

**Affiliations:** Surgeon. Norwich


### *To the Editors of the Medical and Phyfical Journal.*

GENTLEMEN,

THE following cafe of extra uterine fœtus, efpecially as it is attended with fome circumftances tending to the relief of others (who may happen to be in a fimilar fituation) at an earlier period, may not be deemed unworthy of a place in your ufeful publication.

Mrs. COOPER, of Lakenham, fent for a midwife on the 25th of December, 1798, who informed her, fhe would be delivered in a very fhort time, and caufed her great pain, with a fmall difcharge of blood, which continued for fome days; I fuppofe, by endeavouring to rupture the membranes, miftaking the vagina, preffed down before the head of the child, for the membranes.

She was, at that time, at the full period of her reckoning. On the 7th of January, 1799, I was fent for, and as fhe had no pain, I waited a confiderable time, and on examining her, found a globular fubftance very low in the pelvis, which I fuppofed to be the head of the child, but I could not difcover the os uteri. I ftaid with her fome time longer, and told her nothing could be done; but defired they would fend for me if her labour came on. She was at that time paft what fhe reckoned her full time, had bore children before, and had been accurate in her reckonings. Said fhe had felt the child, but thought it different from her former pregnancies, and had felt nothing of the child fince Chriftmas day.

The very deep fnow of 1799 falling foon after this time, rendered the roads from Norwich to Lakenham impaffable for fome days, and I thought fhe had been obliged to call in fuch affiftance as could be procured in the town, till I was informed by one of her relations, whom I attended, that fhe was very unwell, but not delivered. I therefore called upon her on my way to Caiftor, as I thought there muft be fomething fingular in the cafe. She told me, fhe had felt nothing of the child fince Chriftmas day; but, that fhe was certain fhe had previous to that time, although different from her fenfations on fimilar occafions. The body had nearly the fame appearance as in natural pregnancy, with an unevenefs a little above the os pubis. The whole had not exactly the ufual globular form of the impregnated uterus. She had, at this time, exceeded her reckoning more than two months. I found the child's head preffing down very low, and could not difcover the os tincæ in its ufual fituation, but thought I difcovered it above the os pubis. On endeavouring to pafs the finger towards the facrum (the ufual fituation of of the mouth of the uterus where it lies high), it could not paſs, owing to the vagina obſtructing it in every direction backwards. I could paſs the finger very high by the pubis, in which ſituation I found the os uteri as before deſcribed. I mentioned my ſuſpicions of its being an extra-uterine fœtus to Mr. Cooper, ſurgeon of the third Lincoln militia, and requeſted him to ſee her with me: he thought it was the os uteri above the pubis, which could not be felt very diſtinctly, as it was ſituated very high. I examined her again, and concluded it was an extra-uterine fœtus, lying between the rectum and the womb, preſſing the uterus up againſt, and chiefly above the pubis.

Mr. Rigby (whoſe opinions upon theſe ſubjects are much to be reſpected) having ſeen her in the earlier part of her pregnancy, I mentioned the caſe to him, and aſked him to ſee her with me. I was prevented being preſent at the time appointed, but requeſted he would examine her; his opinion was, that there was ſomething extraordinary in the caſe, but was not fully confirmed that it was extra-uterine.

Her health was very much impaired, being affected with diarrhœa, for which, ſhe occaſionally took opiates and aſtringents. I was ſent for, early in the morning, on the third of May, ſhe being now, more than four months paſt her reckoning. I found her very weak and low, her mouth ſore, pulſe quick, and the diarrhœa continued; ſhe had ejected during the night, a conſiderable quantity of fœtid, bloody water. On examining her, I found an opening unlike the os uteri, and my finger paſſed immediately into the head of the child; ſhe had no pain except what I gave her, as I uſed ſome force, preſſing upon the inſide of the bones of the cranium, and endeavouring to dilate the opening. I left her, and told her I would call again, which I did, and took Mr. Rigby with me, who examined and brought away a portion of the cerebrum, which was very offenſive. I afterwards brought away one of the parietal and the occipital bone, and alſo one of the temporal; ſhe was very much exhauſted and faint; we therefore left her, fearing it would be impoſſible to extract the whole of the fœtus. Mr. Rigby called on me on the fourth, and after ſome converſation, I ſaw Mrs. Cooper, and found the other parietal bone in the ſituation it was left on the third, or nearly ſo, which I with difficulty brought away. By introducing my fingers into the opening in the vagina, and fixing them upon the vertebræ of the neck, I brought two of them away; but finding the ſhoulders obſtructed by a part of the vagina, I puſhed my hand paſt it, got my finger into the arm-pit, and at laſt, ſucceeded in bringing away the remaining part of the fœtus, in a highly putrid ſtate, no portion of the navel ſtring remaining. It appeared to be a male child, at full time when it died, both from the formation of the bones, and the ſize of the fœtus: the woman was ſo faint and exhauſted, that I thought it more prudent to deſiſt from introducing my hand to examine for the attachment of the placenta, concluding, that the leaſt evil would be, to truſt to nature for its expulſion, if it was not already diſſolved, and in a ſtate to come away with the diſcharge.

On the fifth I called, with Mr. Aldhouse, and found her very low and faint; ſhe had purged, and her mouth was covered with aphthæ, the diſcharge conſiderable and very offenſive; the womb was nearly in the ſituation before deſcribed, but lower, the opening in the vagina, through which the fœtus was extracted, extending nearly to the neck of the uterus; we could now diſtinguiſh the neck of the uterus, and the uterus itſelf, by the touch; the finger paſſing backwards into the large cavity from which the child was extracted. There was no doubt of a communication between the bowel and the cavity, as ſome ſeeds from a cake, eaten the day before, came away on Mr. Aldhouse's fingers, with a portion of fœces; ſome fœces paſſing likewiſe daily by the vagina, although ſhe had a natural evacuation every day. She remained in the greateſt danger for ſome time; her mouth very ſore; purging at times; part of the excrements paſſing by the opening in the vagina, and part naturally by the rectum. Her plan of medicine was cordials, aſtringents, and opiates, as occaſion required, with wine and nouriſhing ſoups, as the ſtomach would bear them.

She is at this time, Auguſt 16, 1799, able to manage her domeſtic affairs; ſhe paſſes her ſtools naturally, but is obliged to wear a cloth, as ſome fœces paſs by the opening in the vagina: the quantity which paſſes the latter way, being much leſſened, within the laſt month, makes one entertain hopes that the opening into the bowel may cloſe.

There are a ſufficient number of caſes on record of extra-uterine fœtus, both in Engliſh and French authors; and to render uſeful the publication of ſuch occurrences, ſome rules might perhaps be eſtabliſhed for the relief of thoſe who may labour under ſuch extraordinary caſes, at the earlieſt period poſſible.

Great part of the diſtreſs and danger ſeems to ariſe from what the conſtitution ſuffers in ridding itſelf of the impediment, and the lodgment of ſo lager a maſs of putrid matter within the body. In the preſent inſtance, the woman, I think, muſt have died before the bones could have been diſcharged, as this opening into the bowels, muſt have been very high, and there could be no natural effort to propel the bones through the vagina.

If a caſe were to occur where the fœtus was ſituated the ſame as in Mrs. Cooper's, would it not be prudent to make an opening in the vagina, ſufficient cient to admit the extraction, by first perforating the head, and extracting by the crotchet and blunt hook; by which means, probably, the opening into the bowels might have been in this case prevented, and the woman not brought into such imminent danger by the putrefaction of the child?

NORWICH, *Aug.* 20, 1799. E. COLMAN, *Surgeon.*

